# Multi-Residue Method for the Analysis of Stilbene Estrogens in Milk

**DOI:** 10.3390/ijms20030744

**Published:** 2019-02-10

**Authors:** Tianzhu Guan, Yonghai Sun, Yongjun Wang, Zhuolin Li, Tiezhu Li, Ligang Hou

**Affiliations:** 1 College of Food Science and Engineering, Jilin University, Changchun 130062, China; guantz16@mails.jlu.edu.cn (T.G.); sunyh@jlu.edu.cn (Y.S.); 2 Institute of Agricultural Resources and Environment, Jilin Academy of Agricultural Sciences, Changchun 130033, China; yjwang2008@cjaas.com (Y.W.); lizljaas@cjaas.com (Z.L.)

**Keywords:** Estrogen receptor α ligand binding domain (ER-LBD), stilbene estrogens, fluorescence polarization, molecular docking

## Abstract

The rapid analysis of stilbene estrogens is crucially important in the environment, food and health sectors, but quantitation of lower detection limit for stilbene estrogens persists as a severe challenge. We herein described a homologous and sensitive fluorescence polarization (FP) assay based on estrogen receptor α ligand binding domain (ER-LBD) to monitor stilbene estrogens in milk. Under optimal conditions, the half maximal inhibitory concentrations (IC_50_) of the FP assay were 9.27 nM, 12.94 nM, and 22.38 nM for hexestrol, dienestrol and diethylstilbestrol, respectively. And the corresponding limits of detection (LOD) values were 2.94 nM, 2.89 nM, and 3.12 nM. Finally, the assay was applied to determine the stilbenes in milk samples where the mean recoveries ranged from 95.76% to 112.78% and the coefficients of variation (CV) below 12.00%. Furtherly, we have focused our study on high cross-reactivity phenomena by using two in silico approaches, including molecular docking analysis and topology analysis. Overall, docking results show that several residues in the hydrophobic pocket produce hydrophobic interactions with the tested drug molecules, which contribute to the stability of their binding. In this paper, we conclude that the FP method is suitable for the rapid detection of stilbenes in milk samples, requiring no expensive analytical equipment or time-consuming sample preparation. This work offers a practical approach that applies bioscience technology in food safety testing and improves analytical speed and laboratory efficiency.

## 1. Introduction

Dietary estrogens, either naturally produced or synthetic, mimic the effects of natural hormones and are regularly consumed by farm animals [1,2]. Among the different kinds of natural or synthetic estrogens employed in agriculture applications, hexestrol, dienestrol, and diethylstilbestrol, structurally similar to synthetic nonsteroidal estrogens, are often collectively referred to as stilbenes [3]. In order to obtain muscle meat at the cheapest cost in the animal husbandry, stilbenes are widely given as feed additive to promote growth in animals or as a treatment for estrogen-deficiency disorders in veterinary medicine [4,5]. Unsurprisingly, these stilbenes had already been found in agricultural effluent, and may eventually gain entrance to the food chain. Stilbenes could also pollute milky food in particular through the relevant supply chain if used in violation of the legislation [6]. In spite of the usefulness of stilbenes in livestock feeding and clinical treatment, concerns over their properties of teratogenic, mutagenic, and carcinogenic to human’s health have attracted many researchers’ attention [7,8]. Therefore, we should attach great importance to the problem of stilbene residues in milk, and identify the safety of related foodstuff.

Since the milk products are major constituents of human diets, the use of stilbenes in the milky food must be put under strict control. For that reason, many analytical procedures must be put in place to ensure safe animal food products on the markets. Currently, a variety of detection techniques, such as HPLC [9], GC-MS [3,10,11], and LC-MS [12,13], have been extensively developed and used. However, the application of the methods was limited to the demanding for skilled personnel, time consuming and a variety of limitations. To solve the issues above mentioned, a competitive format based on estrogen receptor α ligand binding domain (ER-LBD) has been developed. Because of the advantages over traditional instrumental methods, such as easy-to-use, broad-specificity, and simplicity, the receptor based assay has been successfully applied in many residue detections. 

ER α is mainly expressed in different tissues, including reproductive tissues, breast and liver, and plays important roles in various physiological processes. Studies have clearly shown that, ER is linked to prostate cancer, ovarian cancer, and different kinds of metabolic disease [14]. Twelve helixes, based three-layered antiparallel α-helical sandwich conformation of ER-LBD, create a hydrophobic cavity at the narrow end of the domain, which can adjust according to homologous ligand [15].

To obtain different perspectives on bind mode between stilbenes and ER, we studied at both the electronic level and atomic docking level. The binding affinity of the tested drugs can be obtained by both in silico and experimental methods. And the molecular docking tools are applied widely to predict the interaction between ligand and receptor. The molecular docking approach can be used to model an interaction between a ligand and protein at the atomic level. The docking process involves two basic steps: Prediction of multiple structural conformations in a binding pocket, and scoring the pose in order to rank the multiple solutions. On the other hand, the electrostatic potential (ESP) map offers a visually comprehension of the chemical reactive nature of the molecule. Gradually changing colorings on the molecular density surface correspond to the nucleophilic and electrophilic regions, respectively [16]. Nowadays, the electronic level study can give more and more valuable information on tested drugs [17,18]. 

In this study, ER-LBD was used as a recognition element for quantitative determination the hexestrol, diethylstilbestrol, and dienestrol in milk samples. This approach has been tested with standard estrogenic compounds under the optimized parameters and the analytical parameters were obtained. The applicability of the proposed method was further validated by testing target compounds in real milk samples. Detailed theoretical investigations of the electrostatic potential (ESP) and molecular docking analysis were also performed. This work offers a pragmatic approach that applies bio-sensor technology in food safety and also improves analytical quality and laboratory efficiency.

## 2. Results

### 2.1. Competitive Binding Assay

**Figure 1:**
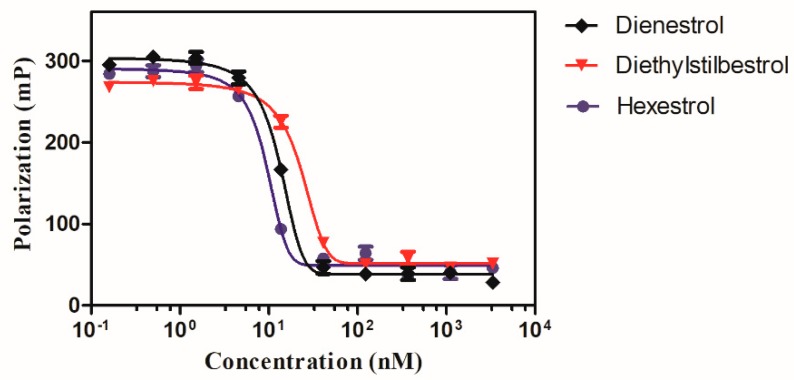
Competitive binding of the tested compounds to estrogen receptor α ligand binding domain (ER-LBD).

The performance of the detection assay may be influenced by many factors, and an optimization process is necessary to improve the sensitivity and reproducibility in follow-up experiments [19]. The strategy of using ER-LBD as a recognition element was promising to produce a broadly specific format. Following validation of the concentration of tracer and recognition element as described in our team work, competitive binding curves for three tested drugs were generated based on their ability to displace the CS and bind to the purified recombinant protein. As shown in Figure 1, all three tested drugs exhibited different replacement ability to the ER-LBD as indicated by their IC_50_ values. The hexestrol exhibited a stronger binding capability for ER-LBD with an IC_50_ value of 9.27 nM compared to that of dienestrol with 12.94 nM and diethylstilbestrol with 22.38 nM.

### 2.2. Determination of Analytical Parameters

Besides the results of IC_50_ mentioned, in the same tests, based on values of IC_10_, the LOD of the tested compounds were in the same order hexestrol < dienestrol < diethylstilbestrol. And the LOD of all analytes were in the range of 2.89–3.12 nM, indicating the proposed analytical method is capable for the trace levels detection. Furthermore, Table 1 below also shows the results of IC_20_–IC_80_. The detection range was achieved in the concentration range from 5.27 to 13.27 nM, 6.60 to 19.28 nM, and 10.23 to 34.53 nM, for hexestrol, dienestrol, and diethylstilbestrol. 

As can be seen from Table 2, diethylstilbestrol was ranked first at 57.82%, followed by dienestrol at 100.00% and hexestrol at 139.59%. Therefore, in an attempt to explain the observed results, the broad-specifity of 3 widely used compounds were compared by their CR values. From a structural point of view, the three structures are somehow symmetrical and the two structures most similar to the synthetic dienestrol. It can be inferred that a similar phenomenon was attributed to the similarities of three estrogens in molecular structure.

### 2.3. Analysis of Spiked Milk Samples

The complexity of food matrices is one of the common challenges of homogeneous quantitative analysis. That is why it is of interest to try different procedures to see whether they can be removed in detection. For the reliable and reproducible experience results, the organic extraction system could be a decisive factor for the assay. Protein removal is a critical step in pretreatment, because it could cause varying degrees of matrix disturbance [4]. Because of acetonitrile can efficiently precipitate proteins out of different samples, thus, the acetonitrile extraction system was chosen for efficient extraction of stilbenes in this assay. As can be seen from Table 2, the treatment shows relatively high extraction efficiency for three tested drugs. Good recovery values and satisfied CV (%) were obtained, which were greater than 95.00% and 11.00% for all the tested compounds. The results showed that the FP assay for stilbenes determination was stable and repeatable. 

### 2.4. Topology Analyses

**Figure 2:**
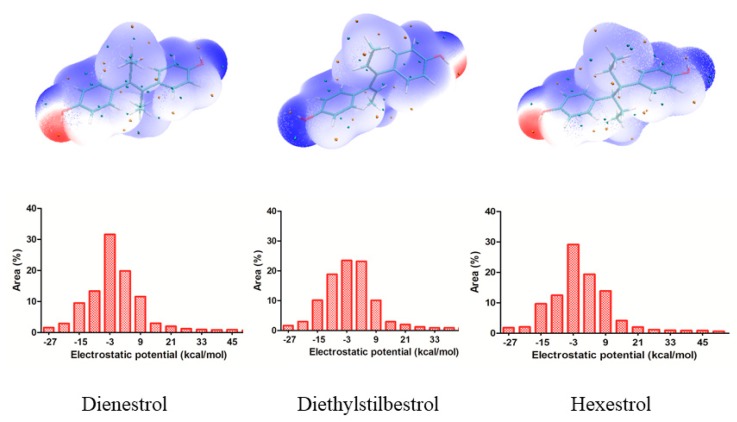
Electrostatic potential (ESP) mapped molecular vdW surface of the dienestrol, diethylstilbestrol and hexestrol. Significant surface local maxima and minima of ESP are labeled by red and blue texts, respectively. The unit is in kcal/mol.

Molecular electrostatic potential (ESP) have been widely used in studying various biological systems and processes [20]. Drug-receptor is one important classes of biological processes in which the initial step is one of “recognition”; the receptor “recognizes” that an approaching molecule has certain key features that will promote their mutual interaction. A knowledge of ESP should therefore help considerably in interpreting its reactive behavior toward charged species and in predicting the beneficial descriptor to anticipate reactive sites for electrophilic and nucleophilic attacks [21,22]. The surface extremes of the dienestrol, diethylstilbestrol and hexestrol are shown in Figure 2 and the graph of surface area plotted against different ESP ranges is also shown in Figure 2. Different values for ESP at the surface are represented by different colors. The red and blue colors represent the positive diffusion region and the negative diffusion territory, respectively.

From ESP map, the negative ESP territory distributes the oxygen atom, which possesses the minimum (−36.95 kcal/mol) of the whole molecular. The sites which possess the minimum points of the isosurface have the electrophilic nature of the oxygen atom and thus are more possibly to be the reactive site. On the other hands, the global maxima of ESP on the dienestrol, diethylstilbestrol and hexestrol appear near the hydrogen atoms. This is because of the presence of oxygen, which attracted lots of electrons from hydrogen atom [23]. It also can be seen that there is a large portion of the molecular surface having negative ESP part, namely from −3 to −27 kcal/mol. There are also small areas having positive ESP value, corresponding to the regions closed to the global ESP minimum and maximum, respectively. Regions of highly negative ESP will clearly accept hydrogen bonds, whereas hydrogen atoms with a positive ESP will be potential donor sites [18]. 

### 2.5. Molecular Docking Analysis

**Figure 3:**
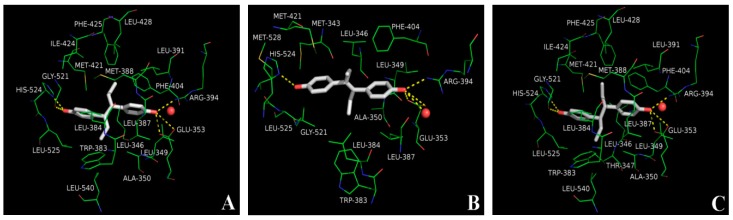
Docking of the tested drug molecules (**A**, Dienestrol; **B**, Diethylstilbestrol; **C**, Hexestrol) to the receptor protein. Red sphere, water molecule; stick, drug molecule; lines, surrounding residues; yellow dashed lines, hydrogen bonds.

Docking results show that several residues in the hydrophobic pocket produce hydrophobic interactions with the tested drug molecules, which contribute to the stability of their binding. The residues of the receptor protein that lie within 4 Å away from the tested drug molecules are shown in Figure 3. As a synthetic agonist ligand, diethylstilbestrol makes hydrogen bonds with GLU353, ARG394, HIS524, and a water molecule (Figure 3B), consistent with the previous report [24]. In addition, both dienestrol (Figure 3A) and hexestrol (Figure 3C) fit into the pocket and adopt a binding mode similar to that of diethylstilbestrol. These two drug molecules make hydrogen bonds with GLU353, ARG394, GLY521, and HIS524. Interestingly, due to the structural similarity of the tested drug molecules, the binding energy of dienestrol, diethylstilbestrol, and hexestrol are close to each other (Table 3).

## 3. Discussion

The estrogen receptors (ERs), members of the steroid receptor superfamily, are ligand-dependent transcription factors and are involved in mutagenic, and carcinogenic to human’s health [25]. The receptor proteins are the natural target of estrogens, such as hexestrol, dienestrol, and diethylstilbestrol, which are structurally similar to synthetic nonsteroidal estrogens. Extensive data have been recently published on estrogens disrupt the endocrine processes in humans and other species, severely affecting reproduction and growth. Therefore, there is an urgent need for a convenient analytical method for screening hexestrol, dienestrol, and diethylstilbestrol in milk samples, which can counteract environmental contaminants and improve public health. 

For a multi-residues analysis system, the utilization of ER-LBD provides a versatile strategy for the stilbenes determinations. In order to evaluate the binding ability of stilbenes, in vitro FP assays using recombinant ER-LBD were performed. Dienestrol efficiently bind to the receptor in FP assay with an IC_50_ of 12.94 nM which is comparable to the diethylstilbestrol (22.38 nM) and hexestrol (9.27 nM). Structurally, the ER consists of three domains namely N-terminal DNA binding domain, N-terminal domain, and C-terminal LBD. The LBD folds into twelve helices, forming a ligand binding pocket. Based on the structural biology analysis of the interaction between dietary estrogens and ER-LBD, all the three drugs can fit into the pocket and adopt a quite similar binding mode. Furthermore, due to the structural similarity of the tested drug molecules, the binding energy of the drugs is close to each other. 

The ESP has a peculiar role in the explanation and analysis of molecular recognition and non-covalent molecular interaction in the initial step of bioactive conformation between the receptor-ligand [26,27]. By utilizing the ESP surface, whether the molecular electrostatic potential of the tested structures is negative or positive are self-explanatory. The most negative ESP region is located at either side of the title drugs which means that the hydroxyl portion was orientated adjacent to the key amino acids to make a strong hydrogen bond interaction. The results are in agreement with the previous findings in that the strong electrostatic interaction of the negative potential with key residues will improve the affinity [27]. These results resembled docking study which the hydrogen bonds were formed between the hydroxyl and several residues of the hydrophobic pocket.

In this study, the developed FP assay provided not only sensitive quantification for the simultaneous stilbenes detection, but also higher efficiency in one test. Furthermore, by use of the two in silico tools, molecular docking analysis and topology analysis, the phenomena of high CR are furtherly expounded. All together, these results demonstrate that ER-LBD proved to be a useful tool for the determination of stilbenes from milk. This format can be expanded to detect other similar chemical contaminants by replacing the targets of interest. Thus, the electrostatic potential of the drugs can play a significant role in the interaction with ER-LBD, and consequently influence the detection effect.

## 4. Materials and Methods

### 4.1. Materials

Coumestrol (CS), Diethylstilbestrol, dienestrol and hexestrol were purchased from Sigma–Aldrich (St. Louis, MO, USA) and Aladdin (Shanghai, China). All other reagents used were of analytical grade.

### 4.2. Development of Fluorescence Polarization (FP) Assay

To begin with, the multi-residue detection method strongly depends on the broad-spectrum of the recognition element with the tested molecules, including hexestrol, diethylstilbestrol, and dienestrol. Therefore, we need to explore a stabilized preparation method of the broad-spectrum receptor, to competitively bind with the tested molecules. To obtain the ER-LBD, the expression of the protein of interest fused with glutathione S-transferase (GST) was produced as already described by [28], and purification of the product by glutathione sepharose (GST) column.

In order to obtain an efficient detection format, the tracer concentration being used should reach a fluorescence intensity that is approximately 5–10-folds the signal of background buffer. And the ER-LBD concentration corresponding to 50% of the maximal signal was chosen as the optimum. Next, the three tested compounds were gradient diluted to yield a 10-point working ligand in a black 96-well plate. And then the native fluorescent phytoestrogen, coumestrol (CS), was added to give a working concentration of 10 nM. Finally, purified ER-LBD was diluted and added to give a final concentration of 250 nM [29]. 

Competitive binding curves were obtained by plotting FP value against the ligand concentration and fitted to a four parameter logistic equation:

Y = {(A − D)/[[1 + X/IC_50_] ^B^} + D (1)

where Y and X correspond to the FP value and the tested compound concentrations, A and D are the asymptotic maximum and asymptotic minimum, respectively, and B is the curve slope at the IC_50_.

The FP value was measured on a FlexStation 3 microplate reader (Molecular Devices, Sunnyvale, CA, USA) with excitation at 355 nm and emission at 405 nm. The obtained polarization values were analyzed by using GraphPad Prism 5 (GraphPad Software, San Diego, CA, USA).

### 4.3. Determination of Analytical Parameters

The competitive binding curves for each corresponding stilbene estrogens were evaluated to obtain the analytical parameters of this assay. The limit of detection (LOD) was defined as IC_10_, which is the ligand concentration of standard inhibiting 10% of tracer binding with the receptor. And the detectable range corresponds to the concentration of ligand varying from IC_20_ to IC_80_, which are the concentrations corresponding to 20% and 80% of the maximal signal. Furthermore, to determine the broad-specifity of this assay, the cross-reactivity (CR) was experimentally calculated by the following equation:

CR = [IC_50_ (dienestrol)/IC_50_ (stilbenes)] × 100% (2)

### 4.4. Sample Treatment

Development of procedures for the determination of estrogenic stilbenes in milk has always been a challenge. Thus, prior to analysis, the proper sample pre-treatments of milk samples are required to evaluate potential matrix effects on the proposed method. Commercial milk samples from a local supermarket were analyzed with spiking concentration for each estrogen. A portion of 5.0 mL of milk was mixed with 10 mL of acetonitrile. The mixtures were vortex mixed for 5 min and left to stand to remove proteins. The extraction with acetonitrile obtained from two extractions were combined and centrifuged for 10 min at 3000 rpm. After the removal of extraction with a vacuum rotary evaporator at 50 °C, the extract was dissolved in 1 ml methanol and filtered through a 0.45 mm membrane filter for analysis.

Method validation was performed by assaying recovery in spiked samples. In order to estimate the precision and accuracy, blank samples were spiked with standard hexestrol, diethylstilbestrol and dienestrol at three different concentrations and assayed as described above. The average recovery and the percent coefficient of variation (CV) was conducted in three experiments with a minimum of three replicated at each concentration. 

### 4.5. Electrostatic Properties

Electrostatic potential (ESP) created in the space around a molecule by its nuclei and electrons is important for studying the molecule’s reactive behavior [21,30]. The ESP has been particularly useful as an indicator of the sites or regions of a molecule to which an approaching electrophile is initially attracted, and it has also been applied successfully to the study of interactions that involve a certain optimum relative orientation of the reactants, such as between a drug and its cellular receptor [22]. A comprehensive study of ESP of stilbenes could be helpful for a deeper understanding of the interaction between stilbenes and receptor. Multiwfn, the quantitative multifunctional wavefunction analysis program, is capable of partitioning the whole vdW surface into multiple fragments, and this feature allows us to discuss the characteristic of ESP distribution of stilbenes [31]. The ESP map was rendered by the VMD program based on the outputs of Multiwfn. Global minima and maxima of ESP are also represented as cyan and orange spheres.

### 4.6. Automated Docking Procedure

The crystal structure of hERα-LBD in complex with diethylstilbestrol was downloaded from the Protein Data Bank (ID: 3ERD) [24]. The initial structures of the tested drug molecules were constructed with GaussView and optimized with Gaussian 09W [32]. The automated docking was performed with AutoDockTools to explore the binding modes between hERα-LBD and drug molecules [33]. The binding energy was calculated based on the scoring function. The docking results were visualized with PyMol [34].

## Figures and Tables

**Table 1:** Inhibitory concentrations (IC_50_) values, limits of detection (LOD), and working ranges for three stilbenes in fluorescence polarization (FP) assay (*n* = 3). IC_50_, LOD, IC_20_, IC_80_ values ± standard errors are presented.

Compound	IC_50_ (nM)	LOD (nM)	IC_20_–IC_80_ (nM)	CR (%)
Dienestrol	12.94 ± 0.71	2.89 ± 0.18	6.60 ± 1.07–19.28 ± 0.15	100.00
Diethylstilbestrol	22.38 ±0.81	3.12 ± 0.29	10.23 ± 0.89–34.53 ± 1.79	57.82
Hexestrol	9.27 ± 0.65	2.94 ± 0.13	5.27 ± 1.03–13.27 ± 0.57	139.59

**Table 2:** Recovery and coefficients of variation (CV) of stilbenes in milk (*n* = 3). Recovery ± standard errors are presented.

Compound	Spiked Level (nM)	Recovery (%, *n* = 9)	CV (%)
Dienestrol	8.00	101.39 ± 0.40	10.76
	12.00	106.30 ± 0.70	7.87
	16.00	95.76 ± 1.00	5.10
Diethylstilbestrol	15.00	98.15 ± 0.30	7.58
	20.00	102.61 ± 0.30	10.93
	30.00	104.56 ± 0.30	9.27
Hexestrol	6.00	106.30 ± 0.30	11.86
	10.00	112.78 ± 0.40	9.53
	14.00	96.27 ± 0.40	9.83

**Table 3:** The hydrogen bonds, hydrophobic contacts, and binding energy of the tested drug molecules.

Drug Molecule	Structure	Hydrogen Bonds	Hydrophobic Contacts	Binding Energy (kcal/mol)
Dienestrol	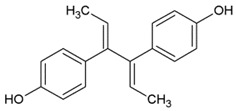	GLU353, ARG394, GLY521, HIS524	LEU346, LEU349, ALA350, TRP383, LEU384, LEU387, MET388, LEU391, PHE404, MET421, ILE424, PHE425, LEU428, LEU525, LEU540	−8.99
Diethylstilbestrol	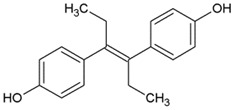	GLU353, ARG394, HIS524, H_2_O	MET343, LEU346, LEU349, ALA350, TRP383, LEU384, LEU387, PHE404, MET421, LEU525, MET528	−9.13
Hexestrol	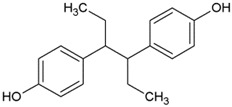	GLU353, ARG394, GLY521, HIS524	LEU346, LEU349, ALA350, TRP383, LEU384, LEU387, MET388, LEU391, PHE404, MET421, ILE424, PHE425, LEU428, LEU525, LEU540	−8.57

## Abbreviations

FP	fluorescence polarization
ER-LBD	estrogen receptor α ligand binding domain
IC_50_	half maximal inhibitory concentrations
LOD	limits of detection
CV	coefficients of variation
ESP	electrostatic potential
GST	glutathione S-transferase
CS	coumestrol
CR	cross-reactivity
